# Metabolic Rewiring by Human Placenta-Derived Mesenchymal Stem Cell Therapy Promotes Rejuvenation in Aged Female Rats

**DOI:** 10.3390/ijms23010566

**Published:** 2022-01-05

**Authors:** Kyeoung-Hwa Kim, Kyung-Ah Lee

**Affiliations:** Department of Biomedical Science, Institute of Reproductive Medicine, College of Life Science, CHA University, Pangyo-ro 335, Seongnam-si 13488, Gyeonggi-do, Korea; khkim@chamc.co.kr

**Keywords:** aging, antiaging, stem cell therapy, metabolism, predictor of rejuvenation, serotonin, hepatocyte proliferation

## Abstract

Aging is a degenerative process involving cell function deterioration, leading to altered metabolic pathways, increased metabolite diversity, and dysregulated metabolism. Previously, we reported that human placenta-derived mesenchymal stem cells (hPD-MSCs) have therapeutic effects on ovarian aging. This study aimed to identify hPD-MSC therapy-induced responses at the metabolite and protein levels and serum biomarker(s) of aging and/or rejuvenation. We observed weight loss after hPD-MSC therapy. Importantly, insulin-like growth factor-I (IGF-I), known prolongs healthy life spans, were markedly elevated in serum. Capillary electrophoresis-time-of-flight mass spectrometry (CE-TOF/MS) analysis identified 176 metabolites, among which the levels of 3-hydroxybutyric acid, glycocholic acid, and taurine, which are associated with health and longevity, were enhanced after hPD-MSC stimulation. Furthermore, after hPD-MSC therapy, the levels of vitamin B6 and its metabolite pyridoxal 5′-phosphate were markedly increased in the serum and liver, respectively. Interestingly, hPD-MSC therapy promoted serotonin production due to increased vitamin B6 metabolism rates. Increased liver serotonin levels after multiple-injection therapy altered the expression of mRNAs and proteins associated with hepatocyte proliferation and mitochondrial biogenesis. Changes in metabolites in circulation after hPD-MSC therapy can be used to identify biomarker(s) of aging and/or rejuvenation. In addition, serotonin is a valuable therapeutic target for reversing aging-associated liver degeneration.

## 1. Introduction

Aging is an elaborate and inevitable process of natural change that affects everyone. To date, there are many theories about aging, and no one theory can fully explain the aging process [1]. However, these theories are important keys for understanding why and how aging occurs. Theories about human aging are generally classified as developmental, psychosocial and biological theories. The developmental theories of aging assert that life is divided into eight stages, with the final stage including people aged 65 and older [2]. Each stage has a developmental crisis or task. According to this theory, progressively achieving competence, skills and greater maturity at each stage can improve quality of life [3]. The psychosocial theories of aging attempt to explain behavior, roles, culture, individual mental health, and relationships associated with increasing age [4]. This theory serves as a framework to explain the increased complexity that is observed with human aging [5]. Biological theories about aging are divided into two major categories: programmed theories and damage or error theories [6]. The programmed theories argue that aging is genetically programmed to occur with biological timelines according to age [6]. The damage or error theories suggest that aging is caused by gradual environmental deterioration and damage to the body’s cells, tissues, and organs [6].

Many theories have been proposed to explain the aging process, there is no consensus on this topic. As life expectancy has dramatically increased, aging and age-related diseases have a large negative impact on human society. Recently, research into longevity and healthy aging has therefore rapidly progressed [7].

Metabolites play a crucial role in aging processes [8]. Hence, metabolomics may offer therapeutic targets and biomarkers for disease and aging [9,10]. Metabolome analysis, which involves measuring the abundance of metabolites, is a useful tool for the diagnosis of disease development and monitoring of aging progression [11]. Blood is a primary carrier of all molecules in the body that provides a rich source of information about metabolites [12]. Blood from elderly individuals has altered antioxidants, nutrients, and compounds required for body activities and organ functions, such as renal function and liver function [13]. Multiple studies have tried to determine the age-related metabolic features in the blood, especially plasma and serum [14,15].

According to previous studies, human placenta-derived mesenchymal stem cell (hPD-MSC) therapy was shown to have therapeutic effects on visual activity in H_2_O_2_-injured rat retinas, hepatic function in a cirrhotic rat model, and regeneration of damaged liver in a TAA-injured rat model [16,17,18]. Recently, many studies in mice, rats, rabbits, and humans have established that transplantation of mesenchymal stem cells (MSCs) from various sources alleviates female reproductive disorders and improves female fertility via increased ovarian function [19,20,21,22,23,24]. In particular, hPD-MSC therapy accelerates ovarian function by activating antioxidant factors and vascular remodeling in young ovariectomized (ovx) rats [25,26]. We also reported the positive effects of hPD-MSC therapy on ovarian function in aged rat ovaries, as indicated by the dramatically increased number of primary follicles obtained via primordial-to-primary follicle transition, which involves circulating miR-145 and BMP-SMAD signaling [21]. While positive effects of hPD-MSCs have been shown in various systems, the underlying mechanisms of the antiaging effects of hPD-MSCs in naturally aged rats have not been extensively studied. After we observed the positive effect of hPD-MSCs on ovarian function, we decided to evaluate whether MSCs also have effects on metabolism in the same animals. Therefore, the aims of this study were to clarify the underlying mechanism of rejuvenation through metabolites after multiple injections of hPD-MSC therapy in old rat models and to discover serum biomarkers that can monitor and predict healthy aging and/or rejuvenation.

## 2. Results

### 2.1. hPD-MSC Therapy Alters Metabolic Patterns in the Advanced-Age Rat Model

**Figure 1:**
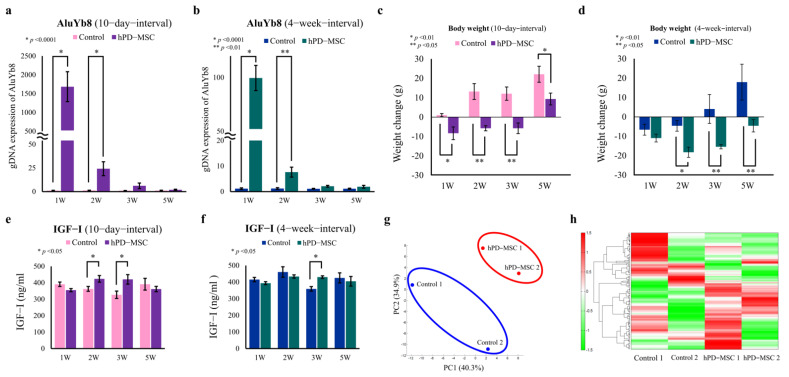
hPD-MSC therapy ameliorates aging-associated phenotypes through numerous metabolic pathways in advanced-age rat models. (**a**,**b**) Analysis of human cells after multiple injections of hPD-MSCs into aged rats by using qRT–PCR. Human DNA (*AluYb8*) expression was detected in the liver after multiple injections of hPD-MSC therapy at 10-day intervals (**a**) or 4-week intervals (**b**). The results show that hPD-MSCs were located in the liver 1–2 weeks after multiple-injection therapy. (**c**,**d**) Changes in body weight after hPD-MSC therapy. Aged rats after multiple injections of hPD-MSC therapy at 10-day intervals or 4-week intervals showed body weight reduction and long-term maintenance. Data are presented as the mean ± SEM. The asterisk represents statistical significance at *p* < 0.05. Control, PBS-injected group; hPD-MSCs, multiple-injection hPD-MSC therapy group. (**e**,**f**) Serum levels of IGF-I, a key regulator of aging, after hPD-MSC therapy. (**g**) PCA of serum metabolite profiles from CE-TOF/MS analysis of aged female rats after hPD-MSC therapy. Serum samples were collected at the 2nd week after three injections of PBS or hPD-MSCs. Control, PBS-injected group; hPD-MSCs, multiple-injection hPD-MSC therapy group. (**h**) Heatmap of the hierarchical cluster analysis of the metabolome analysis after multiple-injection hPD-MSC therapy. The columns indicate the experimental groups of control and multiple-injection therapy at a 10-day interval. The rows show the normalized levels of each metabolite. The dendrogram for each heatmap shows the relatedness of the normalized metabolite level patterns.

To track the fate of hPD-MSCs in the liver, we used qRT–PCR for human-specific Alu sequences. Human DNA (*AluYb8*) sequences were found after multiple injections of hPD-MSC therapy at 10-day intervals and 4-week intervals by tail vein injection. We detected the gDNA expression of *AluYb8* sequences in the liver 1–2 weeks after multiple-injection therapy at both 10-day intervals (Figure 1a) and 4-week intervals (Figure 1b). However, the expression of *AluYb8* was not detected in the control group (Figure 1a,b). Previously, we observed that PKH67-labeled hPD-MSCs were found in the ovaries after multiple-injection therapy [21].

With advanced aging, females have a tendency to gain weight and exhibit metabolic dysfunction. In addition, the age and body weight of animals are closely related to changes in the gene expression of metabolic parameters [27]. Regarding body weight, we found that weight loss was observed in the 10-day-interval group, starting on the first week after multiple cell injections (Figure 1c; pink bar vs. violet bar). At the 5th week in the 10-day-interval group, body weight was slightly increased compared with that at the experimental starting point. However, body weight gain was markedly suppressed in the 10-day-interval group in comparison with the control group (Figure 1c). In addition, aged rats after hPD-MSC therapy displayed significantly lower body weights than those of the control group after 2–5 weeks in the 4-week-interval group (Figure 1d; deep blue bar vs. deep green bar). These results indicate that weight loss was detected earlier in the 10-day-interval group but was maintained long term in the 4-week-interval group.

The IGF-I signaling pathway is a key regulator of aging and is essential for maintaining health span and lifespan [28]. During the aging process, the serum IGF-I concentration declines progressively in humans and mice [28,29]. Thus, we investigated the effect of hPD-MSCs on serum IGF-I levels. Intriguingly, serum IGF-I levels were significantly increased after hPD-MSC therapy at 10-day intervals (Figure 1e) or 4-week intervals (Figure 1f). Previously, we noted that decreased ovarian reserve and ovarian dysfunction caused by aging were markedly improved 2 weeks after multiple-injection therapy at 10-day intervals compared with ovarian reserve and dysfunction in the control group [21]. Taken together, these changes in the ovary, body weight, and serum IGF-I levels indicate that hPD-MSC multiple-injection therapy has a therapeutic effect on the rejuvenation of aging.

On the basis of the concept that a major contributing factor to weight loss is metabolism, we compared the metabolite profiles of serum samples from two aged rats after 2 weeks with or without hPD-MSC stimulation at 10-day intervals. Principal component analysis (PCA) showed obvious dissimilarities between the hPD-MSC therapy group and the control group (Figure 1g; red oval vs. blue oval). Based on the serum samples, 176 peaks were selected to characterize the global metabolic profiles of different groups (Figure 1h). According to the primary metabolic pathway, the major classes identified were lipids and amino acids, carbohydrates, energy, the urea cycle, cofactors and vitamins, nucleotides, and peptides. Supplementary Table S1 lists all the metabolites whose levels were upregulated or downregulated in the serum after multiple injections of hPD-MSC therapy. These results strongly indicate that hPD-MSC therapy alters the metabolic signature in aged animals.

### 2.2. hPD-MSC Therapy Drives Antiaging-Associated Metabolic Changes

**Figure 2:**
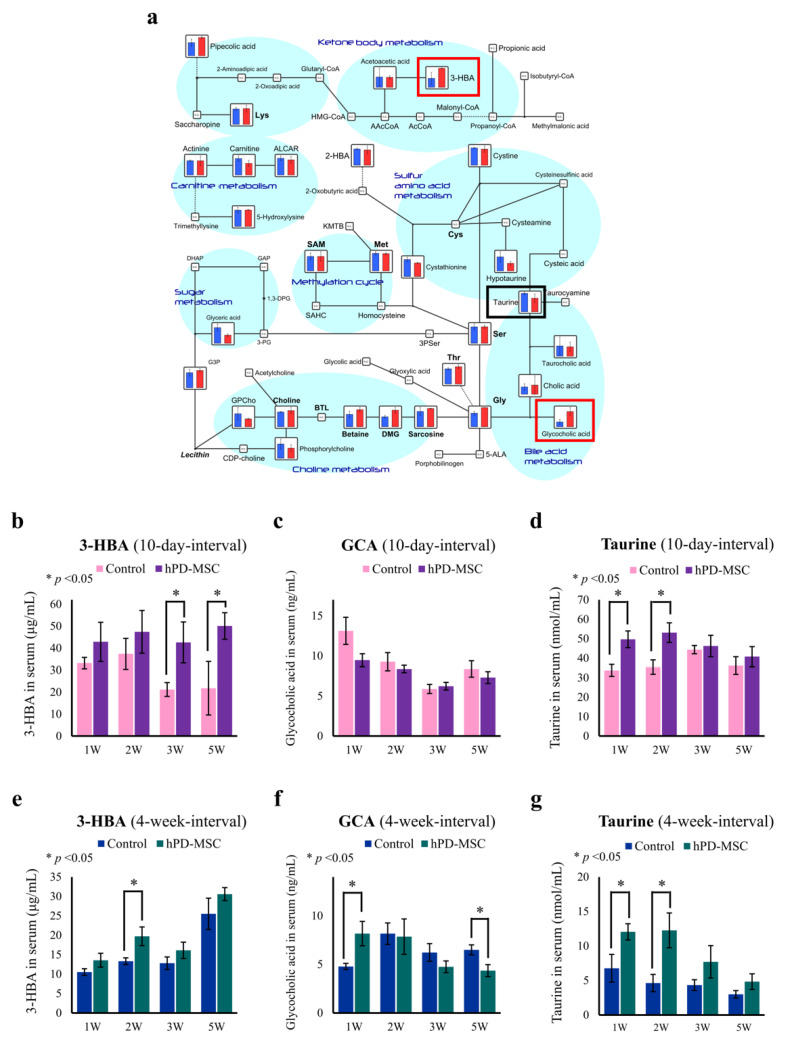
Multiple-injection hPD-MSC therapy is characterized by metabolic changes related to antiaging and extending lifespan. (**a**) The levels of intermediates of lipid and amino acid metabolism are plotted on pathway maps, and the relative quantities of the detected metabolites are shown as bar graphs. Blue bar, control group; red bar, multiple-injection hPD-MSC therapy group. (**b**–**g**) hPD-MSC therapy improves the serum levels of three antiaging metabolites. The levels of 3-HBA (**b**,**e**), glycocholic acid (**c**,**f**), and taurine (**d**,**g**) as determined by ELISA at various time points after multiple injections of hPD-MSC therapy at 10-day (**b**–**d**) or 4-week (**e**–**g**) intervals. Data are presented as the mean ± SEM. The asterisk represents statistical significance at *p* < 0.05. Control, PBS-injected group; hPD-MSCs, multiple-injection hPD-MSC therapy group.

Metabolic regulation dramatically affects the aging process. After we noted that hPD-MSC therapy causes metabolic changes in serum (Figure 1h), we validated the metabolites that play a key role in aging mechanisms, antiaging mechanisms, and lifespan by ELISA. It has been reported that metabolites such as 3-hydroxybutyric acid (3-HBA), glycocholic acid (GCA), and taurine play roles in preventing aging processes, which will increase healthy lifespan [30,31,32]. Based on the metabolite profile by CE-TOF/MS analysis, 3-HBA and GCA were markedly increased in serum after multiple injections of hPD-MSC therapy (Figure 2a, red boxes), whereas taurine was slightly reduced after hPD-MSC therapy (Figure 2a, black boxes). Based on the ELISA results, however, we observed that multiple-injection hPD-MSC therapy showed increased taurine as well as 3-HBA and GCA, anti-aging-related metabolites. The 3-HBA levels increased in the 10-day-interval group and 4-week-interval group compared with the control group (Figure 2b,e). We observed that GCA levels were not significantly changed in the 10-day-interval group (Figure 2c); however, GCA levels were significantly increased in the first week in the 4-week-interval group (Figure 2f). Unlike the metabolomic analysis data, which decreased after therapy, taurine was significantly increased at the first and second weeks after multiple-injection hPD-MSC therapy in both the 10-day- and 4-week-interval groups compared with the control group (Figure 2d,g). Thus, our data show that hPD-MSC therapy resulted in increased serum levels of 3-HBA, GCA, and taurine, which are associated with anti-aging and rejuvenation properties, and suggest that they have essential roles in delaying the aging process and prolonging the healthy lifespan.

### 2.3. hPD-MSC Therapy Enhances the Production of Serotonin during Aging via Vitamin B6 Metabolism

**Figure 3:**
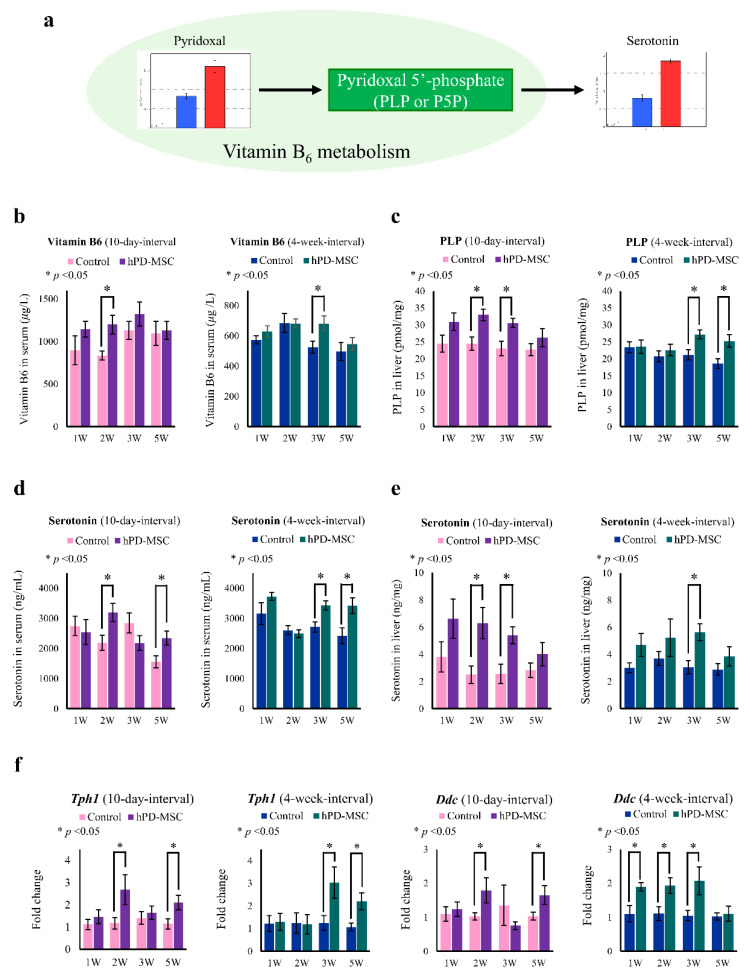
Multiple-injection hPD-MSC therapy induces vitamin B6 metabolism and serotonin synthesis with aging. (**a**) Schematic diagram representing the regulatory pathway between vitamin B6 and serotonin metabolism by CE-TOF/MS analysis. The relative quantities of pyridoxal and serotonin in serum by metabolome analysis are shown as bar graphs. Blue bar, control group; red bar, multiple-injection therapy group. (**b**–**e**) The concentrations of standard metabolites involved in vitamin B6 metabolism and serotonin. Levels of pyridoxal (**b**), PLP (**c**), and serotonin in serum (**d**) and levels of serotonin in the liver (**e**) as determined by ELISA at various time points after multiple injections of hPD-MSC therapy. Pyridoxal, PLP, and serotonin showed higher abundance after multiple-injection hPD-MSC therapy at 10-day intervals or 4-week intervals. Data are presented as the mean ± SEM. The asterisk represents statistical significance at *p* < 0.05. Control, PBS-injected group; hPD-MSCs, multiple-injection hPD-MSC therapy group. (**f**) Changes in the expression of genes related to the production of serotonin were evaluated by qRT–PCR in the liver after multiple injections of hPD-MSC therapy.

Vitamin B6 consists of pyridoxine, pyridoxal, and pyridoxamine, which are cofactors in the tryptophan-serotonin pathway. All forms of vitamin B6 have been detected in serum. In the liver, pyridoxal is converted by pyridoxal kinase into pyridoxal 5′-phosphate (PLP). Importantly, PLP, known as the bioactive form of vitamin B6, is also essential for the synthesis of serotonin [33]. After multiple-injection hPD-MSC therapy at 10-day intervals, we found that the levels of pyridoxal and serotonin were dramatically increased in serum by CE-TOF/MS analysis (Figure 3a and Supplementary Table S1). Thus, we examined the concentrations of vitamin B6 and PLP after hPD-MSC therapy, and the levels of vitamin B6 in serum and PLP in the liver were markedly increased in the multiple-injection therapy groups in both the 10-day-interval and 4-week-interval groups (Figure 3b,c). To determine whether the activation of vitamin B6 metabolism caused by hPD-MSC therapy affected the production of serotonin, the serotonin levels were also measured. As shown in Figure 3d, the serum levels of serotonin in aged rats were relatively high after multiple-injection therapy in both the 10-day-interval and 4-week-interval groups. In parallel, we observed that multiple injections of hPD-MSC therapy improved serotonin levels in the liver (Figure 3e). Interestingly, the levels of vitamin B6, PLP, and serotonin increased faster in the serum and liver in the 10-day interval group than in the 4-week interval group (Figure 3b,e). We also investigated the effect of hPD-MSC therapy on the expression of genes related to serotonin synthesis in the liver. The expression of *tryptophan hydroxylase 1* (*Thp1*) and *dopa decarboxylase* (*Ddc*) was upregulated in the hPD-MSC therapy groups compared with the control group (Figure 3f). These findings strongly suggest that hPD-MSC therapy promotes serotonin production through liver-derived vitamin B6 metabolism and synthesis.

### 2.4. hPD-MSC Therapy-Derived Serotonin Reverses the Failure of Hepatocyte Proliferation Caused by Aging

**Figure 4:**
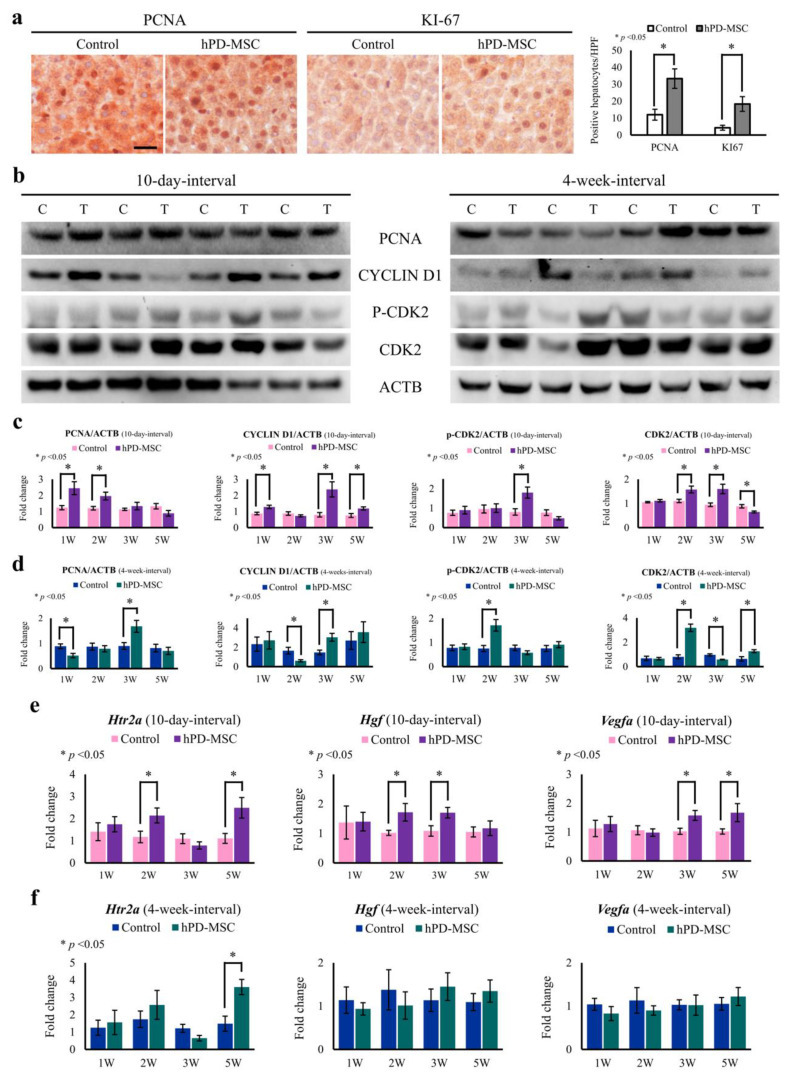
Enhanced hepatocyte proliferation by multiple-injection hPD-MSC therapy can delay liver aging. (**a**) Proliferation of hepatic cells assessed by quantification of immunostaining for the proliferation markers Ki-67 and PCNA in liver specimens after stem cell therapy in advanced-age rat models. Cells were counted in six different sections, and the data are expressed as the number of Ki-67- or PCNA-positive cells ± SEM. The asterisk represents statistical significance at *p* < 0.05. The scale bar indicates 25 μm. (**b**) The levels of the hepatic proteins PCNA, CYCLIN D1, p-CDK2, and CDK2 were evaluated using Western blot analysis at 10-day intervals or 4-week intervals. ACTB was used as a loading control. C, control group; T, multiple-injection hPD-MSC therapy group. (**c**,**d**) Bar graph represents the band intensity of each marker normalized to the intensity of the respective ACTB band at 10-day intervals (**c**) or 4-week intervals (**d**). Data are presented as the mean ± SEM. The asterisk represents statistical significance at *p* < 0.05. Control, PBS-injected group; hPD-MSCs, multiple-injection hPD-MSC therapy group. (**e**,**f**) Changes in the expression of genes related to hepatocyte proliferation in the liver were evaluated by qRT–PCR after multiple injections of hPD-MSC therapy at 10-day intervals (**e**) or 4-week intervals (**f**).

Recent studies on rodents revealed that recovery of impaired liver during aging is controlled by intracellular and extracellular factors [34,35,36]. In the present study, the serum levels of serotonin were enhanced via PLP induced by hPD-MSC therapy. As an increased circulating serotonin concentration is positively associated with liver regeneration [37], our observations suggest that hPD-MSC therapy may be crucial for normal hepatocyte proliferation during the aging process. We then tested whether hPD-MSC therapy-induced serotonin in aged rats correlated with an improvement in hepatocyte proliferation. We assessed the well-established classical biomarkers of cell proliferation (Ki-67 and PCNA) in the liver with or without hPD-MSC therapy. Two weeks after multiple-injection therapy at 10-day intervals, quantification of both markers revealed a dramatic increase in hepatic cell proliferation in aged rats (Figure 4a), indicating that stem cell-derived signaling mediates hepatocyte proliferation with aging. To observe the effect of hPD-MSC therapy on proliferation, we examined the expression of hepatic proteins, such as PCNA, CYCLIN D1, cyclin-dependent kinase 2 (CDK2), and p-CDK2. We found that in both the 10-day-interval (Figure 4b,c) and 4-week-interval groups (Figure 4b,d), the levels of PCNA protein were significantly increased, which was similar to the results obtained with IHC analysis (Figure 4a). As expected, the expression of the hepatic proteins CYCLIN D1, p-CDK2, and CDK2 was significantly enhanced in the 10-day-interval and 4-week-interval groups compared to the control group (Figure 4b–d).

We next examined whether hPD-MSC therapy is related to changes in the expression of the serotonin receptor (*5-hydroxytryptamine receptor 2a*; *Htr2a*) and liver regeneration mediators (hepatocyte growth factor and vascular endothelial growth factor *a*; *Hgf* and *Vegfa*). As a result, hPD-MSC therapy restored the upregulation of *Htr2a* in the liver with aging (Figure 4e,f), indicating that serotonin receptor upregulation is related to the proliferation process. We also found that elevation of *Hgf* and *Vegfa* was observed after multiple-injection hPD-MSC therapy at 10-day intervals (Figure 4e). In contrast, multiple-injection therapy at 4-week intervals had no effect on the expression of *Hgf* and *Vegfa* in aged rats at any time point tested (Figure 4f). These data suggest that hPD-MSC therapy alleviates the aging-associated decline in the capacity of hepatocytes to proliferate.

### 2.5. hPD-MSC Therapy Induces Mitochondrial Biogenesis in the Liver with Aging

**Figure 5:**
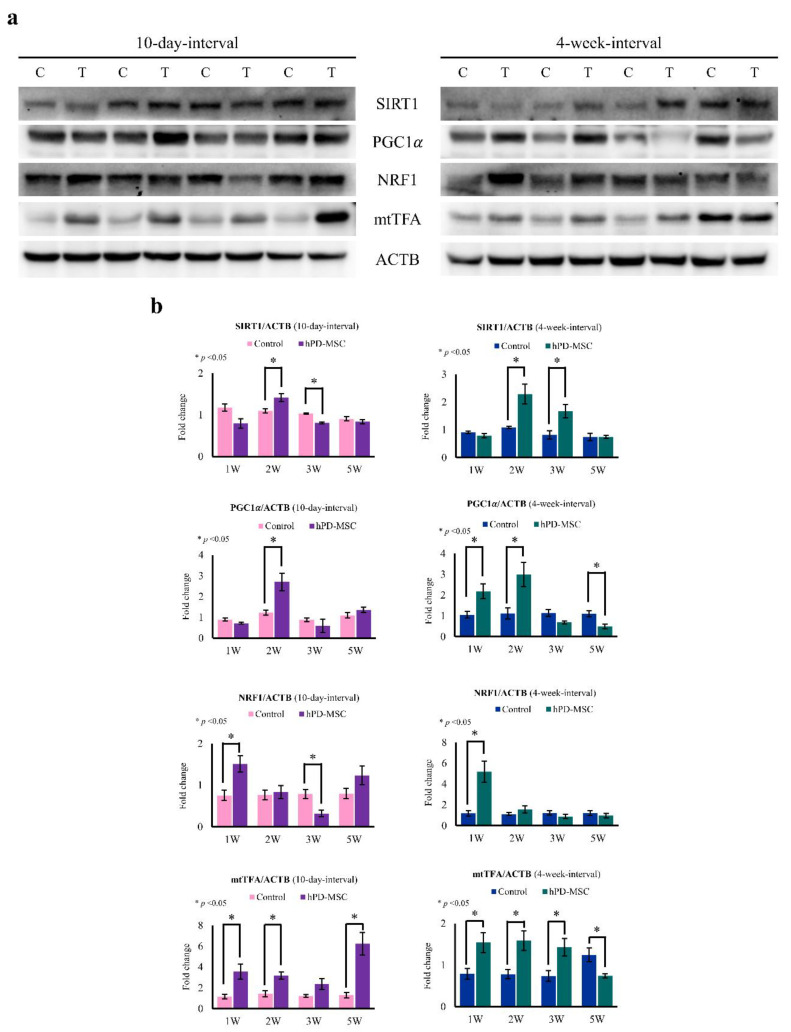
Multiple injections of hPD-MSC therapy enhanced mitochondrial biogenesis in the liver. (**a**) Protein expression of the mitochondrial biogenesis markers SIRT1, PGC1α, NRF1, and mtTFA at 10-day intervals or 4-week intervals was investigated using Western blot analysis. ACTB was used as a loading control. C, control group; T, multiple-injection hPD-MSC therapy group. (**b**) Bar graph represents the band intensity of each marker normalized to the intensity of the respective ACTB band. Data are presented as the mean ± SEM. The asterisk represents statistical significance at *p* < 0.05. Control, PBS-injected group; hPD-MSCs, multiple-injection hPD-MSC therapy group.

Mitochondrial biogenesis occurs during the process of liver regeneration in coordination with cellular proliferation and DNA replication [38]. Suppression of liver regeneration inhibits peroxisome proliferator-activated receptor-γ coactivator 1α (PGC1α) and mitochondrial transcription factor A (mtTFA), which are key factors in the dynamics of mitochondrial biogenesis, resulting in impaired mitochondrial biogenesis [39]. In this study, we observed that hPD-MSC therapy augmented the proliferation of hepatic cells during aging (Figure 4). Therefore, to determine whether hPD-MSC therapy affects mitochondrial biogenesis during hepatocyte proliferation, we used four classical markers of mitochondrial dynamics: sirtuin 1 (SIRT1), PGC1α, nuclear respiration factor 1 (NRF1), and mtTFA. SIRT1 deacetylates PGC1α, and PGC1α activates NRF1, resulting in the induction of mtTFA and stimulation of mitochondrial biogenesis. Western blotting showed that SIRT1 levels were increased in the liver after hPD-MSC therapy (Figure 5a,b). After multiple-injection therapy, the protein levels of PGC1α were increased, indicating enhanced PGC1α activation (Figure 5a,b). Moreover, the expression levels of NRF1 and mtTFA, which are involved in regulating mitochondrial dynamics, were all increased in the liver with aging (Figure 5a,b). In summary, hPD-MSC therapy stimulates mitochondrial biogenesis caused by activation of the SIRT1-PGC1α-NRF1-mtTFA signaling pathway, resulting in increased hepatocyte proliferation in aged rats.

## 3. Discussion

**Figure 6:**
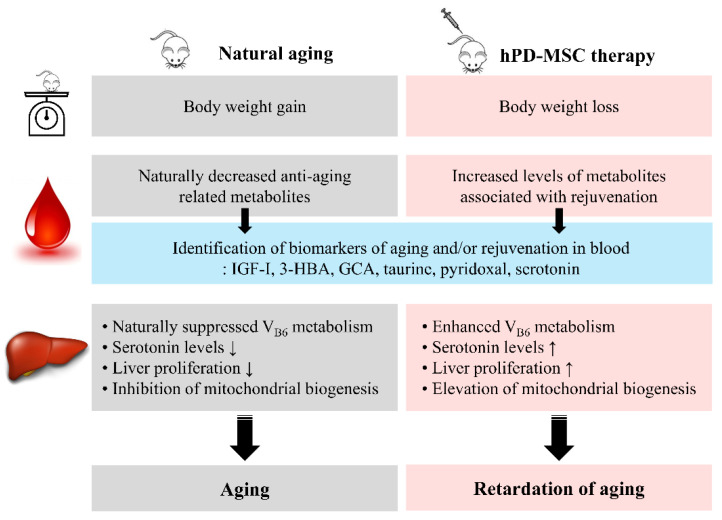
Schematic diagram of the experimental strategy used in the present study to investigate hPD-MSC therapy-induced rejuvenation in aged female rats. After hPD-MSC therapy, aged rats exhibited body weight reduction through metabolic regulation. Changes in factors in the blood related to metabolic alterations after hPD-MSC therapy should be considered candidate biomarker(s) of aging and/or rejuvenation. Furthermore, hPD-MSC therapy promoted serotonin production due to the activation of vitamin B6 metabolism. After hPD-MSC therapy, enhanced liver serotonin production may be essential for hepatocyte proliferation in relation to mitochondrial biogenesis. Clinical application of hPD-MSC therapy in elderly individuals in the future would be a solution for improving quality of life by slowing the aging process.

In this study, the effects of multiple injections of hPD-MSC therapy on aging-associated physiological changes were explored. Our results showed that multiple injections of hPD-MSC therapy dramatically decreased the body weight of aged rats through metabolic regulation. Importantly, we provide novel and specific information on the changes in metabolite levels after hPD-MSC therapy, such as increased IGF-I, 3-HBA, GCA, taurine, PLP, and serotonin. These metabolites are inversely associated with chronological aging. We found hPD-MSCs in old rat liver after multiple-injection therapy at 10-day intervals or 4-week intervals, as we expected, as well as increased hepatocyte proliferation after hPD-MSC therapy. Multiple-injection therapy changed the expression of mRNAs and proteins associated with liver regeneration and mitochondrial biogenesis via increased serotonin caused by activation of vitamin B6 metabolism in the body. Taken together, these results indicate that hPD-MSC therapy has critical effects on preventing the aging process and prolonging healthy lifespan through the control of metabolic dynamics (Figure 6).

It is well known that the most important issue in xenotransplantation for cell therapy is immune compatibility. However, unlike cells from other lineages, the unique characteristics of MSCs, including the low immunogenicity of cells derived from most sources for transplantation (autogeneic, allo-, and third-party MSCs), have already been demonstrated by other scientists [40,41,42,43]. Moreover, some investigators argue that MSCs have immune evasion abilities but are not immune privileged [44]. Although both issues are still under debate and mechanisms underlying immune rejection are still being investigated, we concluded that the present study was conducted in a setting that may prevent direct immune rejection based on HLA alleles without additionally requiring the use of immunosuppressive drugs. According to previous studies, MSCs do not express HLA-II, which is known to be a potent alloantigen, suggesting no relevance to the stimulation of CD4 T cells [45,46]. Although the expression of HLA-I by MSCs may allow their interaction with CD8 T cells, their hypoimmunogenic phenotype was fully validated by the observation of their direct modulation of T cell induction or secretion of immunosuppressive factors [47,48]. We observed that IL-6 expression in the liver was not significantly different between the control and therapy groups (Supplementary Figure S1). In addition, the levels of IL-6 in the ovary markedly decreased at 1 week after hPD-MSC therapy in the 10-day-interval group and at 5 weeks after hPD-MSC therapy in the 4-week-interval group (Supplementary Figure S1). In conjunction with previous findings, allogeneic hPD-MSC therapy did not trigger the immune response in aged rats in this study.

Previously, we found that hPD-MSCs have therapeutic effects on ovarian aging [21]. Accordingly, we thought that hPD-MSC therapy may have positive antiaging effects, and we confirmed that body weight loss after multiple injections of hPD-MSC therapy was tightly linked with metabolic alterations. It has been reported that moderate and progressive weight loss improves metabolic function in the body [49]. In mammals, growth hormone regulates IGF-I levels in serum and tissues. Previous work has established that centenarians (100 years of age) and semi-supercentenarians (≥105 years of age) have a good metabolic profile, characterized by preserved glucose tolerance and insulin sensitivity with low serum IGF-I [50]. In humans, a decline in IGF-I levels has been shown to be associated with a significantly higher incidence of diabetes, osteoporosis, dementia, and Alzheimer’s disease [51,52,53]. In rodents, reductions in serum IGF-I levels during aging impair a healthy lifespan [28]. On the other hand, IGF-I can modulate lifespan in diverse species, including yeast, worms, fruit flies, rodents, and humans [54]. As expected, we found that in aged rats, hPD-MSC therapy led to a reduction in body weight with increased serum IGF-I levels during the aging process. These data provide a hypothetical basis that stem cell therapy could be a more effective method for natural body weight loss without any stressful diets or weight loss pills in elderly individuals, obese individuals, and patients with metabolic syndrome.

Metabolic alterations are fundamental to the aging process [51,55,56,57,58]. In fact, as shown in our results, serum metabolites that increased significantly with hPD-MSC stimulation included factors related to delayed aging and expanded lifespan. According to published studies, 3-HBA is an important molecule that exhibits antiaging effects and can increase longevity by suppressing aging- and oxidative stress-induced changes, such as centrosome amplification, hyperproliferation, and DNA damage accumulation [30,59]. Moreover, cholic acid and chenodeoxycholic acid are produced by the liver in humans and can be conjugated with glycine or taurine to form GCA, taurocholic acid, glycochenodeoxycholic acid (GCDCA), and taurochenodeoxycholic acid [60]. GCA is one of the bile acids that helps emulsify fats and absorb fat-soluble vitamins [61]. In the progeria mouse model, a diet enriched in cholic acid enhanced the healthspan and lifespan through an increase in glycine-conjugated bile acids, including GCA and GCDCA. Taurine is found at higher concentrations in most cells and plays a role in alleviating symptoms of aging [62], and its level gradually decreases with aging [63]. Three metabolites, namely, 3-HBA, GCA, and taurine, are able to attenuate hallmarks of senescence. Our results showed that the concentrations of 3-HBA, GCA, and taurine were significantly increased in the serum after hPD-MSC treatment, which may contribute to its antiaging role. We suggest these metabolites as candidate biomarkers in serum for antiaging, healthy lifespan, and rejuvenation. These specific alterations in the serum metabolites caused by multiple-injection hPD-MSC therapy oppose the naturally occurring metabolic dynamics during aging and have a protective anti-aging role.

Vitamin B6, also known as pyridoxine, pyridoxal, and pyridoxamine, is a water-soluble vitamin that is added to foods and supplements. Pyridoxine is predominant in plants, whereas pyridoxal and pyridoxamine are mainly present in animal tissues [64]. Absorbed vitamin B6 is converted to PLP in the liver. PLP acts as a cofactor and antioxidant molecule in numerous enzymatic reactions, including the metabolism of proteins, carbohydrates, and fats; synthesis of histamine; scavenging of reactive oxygen species (ROS); support of the immune system and brain health; and synthesis and/or catabolism of certain neurotransmitters [65,66]. On the other hand, serotonin is a small molecule linked to antiaging activity that is closely associated with PLP. Intriguingly, PLP-dependent activities regulate metabolic pathways that convert tryptophan to serotonin in the liver [67]. Tryptophan hydroxylase (THP1 and THP2) converts tryptophan to 5-hydroxytryptophan, which in turn is metabolized to serotonin by the PLP-dependent enzyme dopa decarboxylase (DDC) [68]. We showed that serum levels of pyridoxal and serotonin were increased with stem cell therapy. Vitamin B6 is crucial for the tryptophan-serotonin pathway. After multiple injections of hPD-MSC therapy, the increased PLP in the liver via the vitamin B6 metabolic pathway leads to upregulation of serotonin through enhanced expression of genes related to serotonin biosynthesis (*Thp1* and *Ddc*). Therefore, we concluded that hPD-MSCs regulate the levels of serotonin through a PLP-dependent pathway, thereby efficiently safeguarding the health span of elderly individuals.

The liver has an excellent capacity to regenerate after injury or resection, but this unique regenerative ability of the liver is reduced with aging, so it represents a critical problem in elderly patients with liver diseases [69,70]. In other words, aging involves reductions in liver volume, blood flow, immune function, and liver regeneration capacity, which may contribute to an increased risk of liver disease. The liver is mainly composed of hepatocytes. Under both acute and chronic injury conditions, hepatocyte proliferation plays a distinctive role in liver homeostasis and regeneration [71]. Recently, it has been noted that MSCs are important for liver regenerative medicine and have been used in the treatment of liver cirrhosis [72]. Patients with liver cirrhosis are known to have markedly lower serotonin levels than healthy individuals [67]. It is also well known that the concentration of serotonin declines during aging [73,74,75]. In this study, as a result of the increased serotonin levels in serum and liver by vitamin B6 metabolism induced by hPD-MSC treatment, increased hepatocyte proliferation markers such as Ki-67 and PCNA and elevation of hepatic proteins were observed. Changes in serotonin levels after hPD-MSC therapy may cause age-related changes in the hepatocyte proliferation rate. In addition, mitochondria are the main energy source and play a critical role in liver function [76]. In hepatocytes, adequate mitochondrial function is maintained by mitochondrial biogenesis and/or increased enzyme activity. As expected, we observed that the proteins related to mitochondrial proliferation, namely, SIRT1, PCGα1, NRF1, and mtTFA, were substantially enhanced in the aged liver with hPD-MSC treatment compared with the control group. These results collectively suggest that hPD-MSC therapy-derived serotonin might impede the predicted age-related decline in liver proliferation through interaction with mitochondrial biogenesis.

In conclusion, we found that multiple injections of hPD-MSC therapy-induced alterations in serum metabolites, and specifically, the levels of four metabolites (3-HBA, GCA, taurine, and PLP), a growth factor (IGF-I), and a hormone (serotonin), whose levels are inversely correlated with aging, were significantly elevated. Because changes in the levels of these metabolites are involved in the antiaging process, we propose that these metabolites are candidate metabolic markers for predicting aging and rejuvenation. Additionally, hPD-MSC therapy alleviates the symptoms of age-associated health conditions, including body weight loss and liver proliferation, by activating mitochondrial biogenesis. Therefore, we conclude that hPD-MSC therapy would delay the aging process.

## 4. Materials and Methods

### 4.1. Reagents

Reagents were purchased from Sigma-Aldrich (St. Louis, MO, USA) unless otherwise noted.

### 4.2. Culture of hPD-MSCs

hPD-MSCs were kindly provided by Gi Jin Kim (CHA University, Pangyo, Korea). hPD-MSCs were isolated and characterized as reported previously [77,78]. Briefly, hPS-MSCs were cultured in α-MEM containing 10% fetal bovine serum (FBS; Corning Inc., Corning, NY, USA), 1% penicillin-streptomycin (Life Technologies, Carlsbad, CA, USA), 1 mg/mL heparin, and 100 μg/mL FGF4 (PeproTech, Rocky Hill, NJ, USA) with 5% CO_2_ at 37 °C.

### 4.3. Multiple Injection of hPD-MSC Therapy

All procedures were approved by the Institutional Animal Care and Use Committee (IACUC 190163) of the CHA Laboratory Animal Research Center. Sprague–Dawley (SD; 52–54 weeks of age) rats were obtained from Janvier Labs (Le Genest-Saint-Isle, France). These animals were randomly divided into multiple-injection hPD-MSC therapy groups and a control group (*n* = 24 in each group). For multiple-injection hPD-MSC therapy, hPD-MSCs (5 × 10^5^) were injected three times into rats via the tail vein at 10-day intervals or 4-week intervals. The rats in the control group were only injected with phosphate-buffered saline (PBS). The rats were sacrificed 1, 2, 3, and 5 weeks following the transplantation of hPD-MSCs, and organ samples were collected and immediately frozen for further analysis. Serum was collected by centrifugation and stored at −80 °C for metabolome profiling and metabolite assays.

### 4.4. RNA Extraction and Quantitative Real-Time RT–PCR (qRT–PCR)

Total RNA was extracted from the liver using TRIzol (Invitrogen, Carlsbad, CA, USA) reagent and reverse transcribed to generate cDNA using M-MLV Reverse Transcriptase (Promega, Madison, WI, USA). To measure the expression of target genes in the liver after hPD-MSC therapy, qRT–PCR analysis was performed using a CFX96 Touch™ Real-Time PCR Detection System (Bio-Rad, Hercules, CA, USA). The primer sequences are listed in Supplementary Table S2. iQ SYBR Green Supermix PCR reagents (Bio-Rad, Hercules, CA, USA) were used to monitor the fluorescence as amplification. The results and melting curves were assessed using CFX Maestro software (Bio-Rad, Hercules, CA, USA). The expression of each gene was normalized to the expression of *Gapdh*. The relative change in gene expression was calculated using the comparative C_T_ method.

### 4.5. Genome Extraction

Rat genomic DNA was extracted from the liver after hPD-MSC therapy as previously reported [79]. After RNA isolation with 500 μL of TRIzol, 250 μL of back extraction buffer (BEB; 4 M guanidine thiocyanate; 50 mM sodium citrate; 1 M Tris, pH 8.0) was added to the interphase-organic phase mixtures and then incubated at room temperature for 10 min. After centrifugation, the upper phase was removed, 100% isopropanol was added, and the samples were incubated overnight at −80 °C. After centrifugation, the supernatant was removed, and the pellets were washed with 70% ethanol. The pellets were eluted with Tris-EDTA (10 mM Tris; 0.1 mM EDTA, pH 8.0).

### 4.6. Human Alu Sequence and qRT–PCR

We synthesized the primers as described previously by Walker et al. [80]. The *AluYb8* sequences are listed in Supplementary Table S2. qRT–PCR was performed with *AluYb8* primers and 10 ng of genomic DNA from the liver.

### 4.7. Metabolome Profile of Serum by CE-TOF/MS Analysis

Serum metabolomic analysis was performed employing the Basic Scan package from Human Metabolome Technologies (HMT) Inc. (Tsuruoka, Japan) and CE-TOF/MS as previously described [81]. CE-TOF/MS analysis was performed with serum samples from two aged rats after 2 weeks with or without hPD-MSC therapy at 10-day intervals.

### 4.8. Western Blotting

Western blotting was performed using a standard protocol. We used the following primary and secondary antibodies: PCNA (1:1000; ab92552, Abcam, Cambridge, MA, USA), CYCLIN D1 (1:1000; #55506, Cell Signaling Technology, Danvers, MA, USA), phospho-CDK2 (p-CDK2; 1:1000; #2561, Cell Signaling Technology, Danvers, MA, USA), CDK2 (1:1000; #18048, Cell Signaling Technology, Danvers, MA, USA), SIRT1 (1:1000; #9475, Cell Signaling Technology, Danvers, MA, USA), PGC1α (1:1000; NAP1-04676, Novus Biologicals, Littleton, CO, USA), NRF1 (1:1000; #46743, Cell Signaling Technology), mtTFA (1:1000; ab131607, Abcam, Cambridge, MA, USA), ACTIN (1:2000; sc-8432, Santa Cruz Biotechnology, Dallas, TX, USA), ACTB (1:2000; A5316), HRP-conjugated anti-rabbit IgG (1:5000; #65-6120, Invitrogen, Carlsbad, CA, USA) and anti-IgG (1:5000; #62-6520, Invitrogen, Carlsbad, CA, USA). Bands were detected using a ChemiDoc XRD+ system (Bio-Rad, Hercules, CA, USA), and relative intensity was assessed using Image Lab software (Bio-Rad, Hercules, CA, USA).

### 4.9. Serum and Liver Metabolite Levels

Eight different enzyme-linked immunosorbent assay (ELISA) kits were used in the study. For ELISA analysis, one sample of 0.5 mL of serum was obtained from each rat. Liver lysate was prepared by homogenization in ice-cold 0.05 N HCl with a cocktail of protease inhibitors (Thermo Fisher Scientific, Waltham, MA, USA) or PLP assay buffer. The amount of protein in the samples was normalized after quantifying the total protein using the Bio-Rad protein assay dye reagent concentrate. The levels of metabolites in serum or liver at the indicated time points (1, 2, 3, and 5 weeks after multiple-injection hPD-MSC therapy at 10-day intervals or 4-week intervals) were measured using the rat IGF-I Quantikine ELISA kit (MG100, R&D Systems, Minneapolis, MN, USA), rat beta-hydroxybutyric acid ELISA kit (3-HBA; MBS721642, MyBioSource, San Diego, CA, USA), rat glycocholic acid ELISA kit (GCA; MBS7222062, MyBioSource, San Diego, CA, USA), rat taurine ELISA kit (MBS744370, MyBioSource, San Diego, CA, USA), general 5-hydroxytryptamine (5-HT) ELISA kit (MBS2700308, MyBioSource, San Diego, CA, USA), serotonin ELISA kit (ab133053, Abcam, Cambridge, MA, USA), rat vitamin B6 ELISA kit (MBS9711616, MyBioSource, San Diego, CA, USA), and PLP assay kit (ab273312, Abcam, Cambridge, MA, USA). Assays were performed according to the manufacturer’s instructions.

### 4.10. Immunohistochemistry (IHC)

Livers from aged rats after multiple injections of hPD-MSC therapy were fixed in 10% neutral buffered formalin. After dehydration, fixed tissue was embedded in paraffin, sectioned into 8-μm-thick sections, and secured on slides (Pro-beOn Plus, Fisher Scientific, Pittsburgh, PA, USA). Tissue sections were deparaffinized, incubated with 3% H_2_O_2_ in methanol for 20 min, and treated with primary antibodies specific for PCNA (ab92552, Abcam, Cambridge, MA, USA) and Ki-67 (ab16667, Abcam, Cambridge, MA, USA) at a 1:100 dilution overnight at 4 °C. Next, sections were rinsed in PBS and treated with biotinylated secondary antibody (DAKO, Carpenteria, CA, USA) for 20 min at room temperature. Finally, the streptavidin-biotin-peroxidase complex was treated for 25 min at room temperature, and peroxidase activity was visualized with AEC+ staining (DAKO, Carpenteria, CA, USA). Negative control sections were incubated with dilution buffer alone. Hematoxylin was used for counterstaining.

### 4.11. Statistical Analysis

Unless indicated otherwise, all procedures were repeated at least three independent times. The results are expressed as the mean ± SEM. Statistical analyses were performed using Student’s *t*-test (control 10-day-interval group vs. multiple-injection 10-day-interval therapy group; control 4-week-interval group vs. multiple-injection 4-week-interval therapy group), and *p* < 0.05 was considered statistically significant.

## Data Availability

All data are included in the text and Supplementary Materials.

## Supplementary Materials

The following supporting information can be downloaded at: https://www.mdpi.com/article/10.3390/ijms23010566/s1.

## Abbreviations

hPD-MSCs	Human placenta-derived mesenchymal stem cells
IGF-I	Insulin-like growth factor-I
CE-TOF/MS	Capillary electrophoresis-time-of-flight mass spectrometry
MSCs	Mesenchymal stem cells
Ovx	Ovariectomized
PCA	Principal component analysis
3-HBA	3-Hydroxybutyric acid
GCA	Glycocholic acid
PLP	Pyridoxal 5′-phosphate
THP1	Tryptophan hydroxylase 1
DDC	Dopa decarboxylase
CDK2	Cyclin-dependent kinase 2
HTR2A	5-Hydroxytryptamine receptor 2a
HGF	Hepatocyte growth factor
VEGFA	Vascular endothelial growth factor a
PGC1α	Peroxisome proliferator-activated receptor-γ coactivator 1α
mtTFA	Mitochondrial transcription factor A
SIRT1	Sirtuin 1
NRF1	Nuclear respiration factor 1
GCDCA	Glycochenodeoxycholic acid
ELISA	Enzyme-linked immunosorbent assay
5-HT	5-Hydroxytryptamine

## References

[1] Davidovic M., Sevo G., Svorcan P., Milosevic D.P., Despotovic N., Erceg P. (2010). Old age as a privilege of the selfish ones. Aging Dis..

[2] Erikson E.H., Erikson J.M. (1997). The Life Cycle Completed.

[3] Villar F. (2012). Successful ageing and development: The contribution of generativity in older age. Ageing Soc..

[4] Burbank P.M. (1986). Psychosocial theories of aging: A critical evaluation. ANS Adv. Nurs. Sci..

[5] Poon L.W., Martin P., Bishop A., Cho J., da Rosa G., Deshpande N., Hensley R., Macdonald M., Margrett J., Randall G.K. (2010). Understanding centenarians’ psychosocial dynamics and their contributions to health and quality of life. Curr. Gerontol. Geriatr. Res..

[6] Jin K. (2010). Modern biological theories of aging. Aging Dis..

[7] He S., Sharpless N.E. (2017). Senescence in health and disease. Cell.

[8] Lopez-Otin C., Galluzzi L., Freije J.M.P., Madeo F., Kroemer G. (2016). Metabolic control of longevity. Cell.

[9] Clish C.B. (2015). Metabolomics: An emerging but powerful tool for precision medicine. Cold Spring Harb. Mol. Case Stud..

[10] Trivedi D.K., Hollywood K.A., Goodacre R. (2017). Metabolomics for the masses: The future of metabolomics in a personalized world. New Horiz. Transl. Med..

[11] Zhang A., Sun H., Yan G., Wang P., Wang X. (2015). Metabolomics for biomarker discovery: Moving to the clinic. BioMed Res. Int..

[12] Psychogios N., Hau D.D., Peng J., Guo A.C., Mandal R., Bouatra S., Sinelnikov I., Krishnamurthy R., Eisner R., Gautam B. (2011). The human serum metabolome. PLoS ONE.

[13] Chaleckis R., Murakami I., Takada J., Kondoh H., Yanagida M. (2016). Individual variability in human blood metabolites identifies age-related differences. Proc. Natl. Acad. Sci. USA.

[14] Banerjee C., Ulloor J., Dillon E.L., Dahodwala Q., Franklin B., Storer T., Sebastiani P., Sheffield-Moore M., Urban R.J., Bhasin S. (2011). Identification of serum biomarkers for aging and anabolic response. Immun. Ageing.

[15] Kwak J.Y., Hwang H., Kim S.K., Choi J.Y., Lee S.M., Bang H., Kwon E.S., Lee K.P., Chung S.G., Kwon K.S. (2018). Prediction of sarcopenia using a combination of multiple serum biomarkers. Sci. Rep..

[16] Kim J.Y., Park S., Park H.J., Kim S.H., Lew H., Kim G.J. (2021). PEDF-mediated mitophagy triggers the visual cycle by enhancing mitochondrial functions in a H2O2-injured rat model. Cells.

[17] Na J., Song J., Kim H.H., Seok J., Kim J.Y., Jun J.H., Kim G.J. (2020). Human placenta-derived mesenchymal stem cells trigger repair system in TAA-injured rat model via antioxidant effect. Aging.

[18] Kim J.Y., Choi J.H., Jun J.H., Park S., Jung J., Bae S.H., Kim G.J. (2020). Enhanced PRL-1 expression in placenta-derived mesenchymal stem cells accelerates hepatic function via mitochondrial dynamics in a cirrhotic rat model. Stem Cell Res. Ther..

[19] Esfandyari S., Chugh R.M., Park H.S., Hobeika E., Ulin M., Al-Hendy A. (2020). Mesenchymal stem cells as a bio-organ for treatment of female infertility. Cells.

[20] Yang Y., Lei L., Wang S., Sheng X., Yan G., Xu L., Liu J., Liu M., Zhen X., Ding L. (2019). Transplantation of umbilical cord-derived mesenchymal stem cells on a collagen scaffold improves ovarian function in a premature ovarian failure model of mice. Vitr. Cell. Dev. Biol. Anim..

[21] Kim K.H., Kim E.Y., Kim G.J., Ko J.J., Cha K.Y., Koong M.K., Lee K.A. (2020). Human placenta-derived mesenchymal stem cells stimulate ovarian function via miR-145 and bone morphogenetic protein signaling in aged rats. Stem Cell Res. Ther..

[22] Abd-Allah S.H., Shalaby S.M., Pasha H.F., El-Shal A.S., Raafat N., Shabrawy S.M., Awad H.A., Amer M.G., Gharib M.A., El Gendy E.A. (2013). Mechanistic action of mesenchymal stem cell injection in the treatment of chemically induced ovarian failure in rabbits. Cytotherapy.

[23] Ding L., Yan G., Wang B., Xu L., Gu Y., Ru T., Cui X., Lei L., Liu J., Sheng X. (2018). Transplantation of UC-MSCs on collagen scaffold activates follicles in dormant ovaries of POF patients with long history of infertility. Sci. China Life Sci..

[24] Singh N., Mohanty S., Seth T., Shankar M., Bhaskaran S., Dharmendra S. (2014). Autologous stem cell transplantation in refractory Asherman’s syndrome: A novel cell-based therapy. J. Hum. Reprod. Sci..

[25] Seok J., Park H., Choi J.H., Lim J.Y., Kim K.G., Kim G.J. (2020). Placenta-derived mesenchymal stem cells restore the ovary function in an ovariectomized rat model via an antioxidant effect. Antioxidants.

[26] Cho J., Kim T.H., Seok J., Jun J.H., Park H., Kweon M., Lim J.Y., Kim G.J. (2021). Vascular remodeling by placenta-derived mesenchymal stem cells restores ovarian function in ovariectomized rat model via the VEGF pathway. Lab. Invest..

[27] Ghasemi A., Jeddi S., Kashfi K. (2021). The laboratory rat: Age and body weight matter. EXCLI J..

[28] Gong Z., Tas E., Muzumdar R. (2014). Humanin and age-related diseases: A new link?. Front. Endocrinol..

[29] Daughaday W.H., Rotwein P. (1989). Insulin-like growth factors I and II. Peptide, messenger ribonucleic acid and gene structures, serum, and tissue concentrations. Endocr. Rev..

[30] Edwards C., Copes N., Bradshaw P.C. (2015). D-ss-hydroxybutyrate: An anti-aging ketone body. Oncotarget.

[31] Barcena C., Quiros P.M., Durand S., Mayoral P., Rodriguez F., Caravia X.M., Marino G., Garabaya C., Fernandez-Garcia M.T., Kroemer G. (2018). Methionine restriction extends lifespan in progeroid mice and alters lipid and bile acid metabolism. Cell Rep..

[32] Dawson R., Eppler B., Patterson T.A., Shih D., Liu S. (1996). The effects of taurine in a rodent model of aging. Adv. Exp. Med. Biol..

[33] Cellini B., Montioli R., Oppici E., Astegno A., Voltattorni C.B. (2014). The chaperone role of the pyridoxal 5’-phosphate and its implications for rare diseases involving B6-dependent enzymes. Clin. Biochem..

[34] Pibiri M. (2018). Liver regeneration in aged mice: New insights. Aging.

[35] Mde L.B.S., Matias J.E., Montibeller G.R., Siqueira L.C., Nunes Eda S., Grassi C.A. (2006). Effect of aging on liver regeneration in rats. Acta Cir. Bras..

[36] Schmucker D.L., Sanchez H. (2011). Liver regeneration and aging: A current perspective. Curr. Gerontol. Geriatr. Res..

[37] Lesurtel M., Graf R., Aleil B., Walther D.J., Tian Y., Jochum W., Gachet C., Bader M., Clavien P.A. (2006). Platelet-derived serotonin mediates liver regeneration. Science.

[38] Khiati S., Baechler S.A., Factor V.M., Zhang H., Huang S.Y., Dalla Rosa I., Sourbier C., Neckers L., Thorgeirsson S.S., Pommier Y. (2015). Lack of mitochondrial topoisomerase I (TOP1mt) impairs liver regeneration. Proc. Natl. Acad. Sci. USA.

[39] Han L.H., Dong L.Y., Yu H., Sun G.Y., Wu Y., Gao J., Thasler W., An W. (2015). Deceleration of liver regeneration by knockdown of augmenter of liver regeneration gene is associated with impairment of mitochondrial DNA synthesis in mice. Am. J. Physiol. Gastrointest. Liver Physiol..

[40] Zhu S.F., Hu H.B., Xu H.Y., Fu X.F., Peng D.X., Su W.Y., He Y.L. (2015). Human umbilical cord mesenchymal stem cell transplantation restores damaged ovaries. J. Cell. Mol. Med..

[41] Wang Z., Wang Y., Yang T., Li J., Yang X. (2017). Study of the reparative effects of menstrual-derived stem cells on premature ovarian failure in mice. Stem Cell Res. Ther..

[42] Yang M., Lin L., Sha C., Li T., Zhao D., Wei H., Chen Q., Liu Y., Chen X., Xu W. (2020). Bone marrow mesenchymal stem cell-derived exosomal miR-144-5p improves rat ovarian function after chemotherapy-induced ovarian failure by targeting PTEN. Lab. Invest..

[43] Xiao G.Y., Liu I.H., Cheng C.C., Chang C.C., Lee Y.H., Cheng W.T., Wu S.C. (2014). Amniotic fluid stem cells prevent follicle atresia and rescue fertility of mice with premature ovarian failure induced by chemotherapy. PLoS ONE.

[44] Ankrum J.A., Ong J.F., Karp J.M. (2014). Mesenchymal stem cells: Immune evasive, not immune privileged. Nat. Biotechnol..

[45] Lee J.E., Lee J.Y., Park C.H., Eum J.H., Jung S.K., Han A.R., Seol D.W., Lee J.S., Shin H.S., Im J.H. (2020). Cryopreserved human oocytes and cord blood cells can produce somatic cell nuclear transfer-derived pluripotent stem cells with a homozygous HLA type. Stem Cell Rep..

[46] Krampera M., Glennie S., Dyson J., Scott D., Laylor R., Simpson E., Dazzi F. (2003). Bone marrow mesenchymal stem cells inhibit the response of naive and memory antigen-specific T cells to their cognate peptide. Blood.

[47] Beyth S., Borovsky Z., Mevorach D., Liebergall M., Gazit Z., Aslan H., Galun E., Rachmilewitz J. (2005). Human mesenchymal stem cells alter antigen-presenting cell maturation and induce T-cell unresponsiveness. Blood.

[48] Ranganath S.H., Levy O., Inamdar M.S., Karp J.M. (2012). Harnessing the mesenchymal stem cell secretome for the treatment of cardiovascular disease. Cell Stem Cell.

[49] Longo M., Zatterale F., Naderi J., Parrillo L., Formisano P., Raciti G.A., Beguinot F., Miele C. (2019). Adipose tissue dysfunction as determinant of obesity-associated metabolic complications. Int. J. Mol. Sci..

[50] Giuliani C., Garagnani P., Franceschi C. (2018). Genetics of human longevity within an eco-evolutionary nature-nurture framework. Circ. Res..

[51] Barzilai N., Huffman D.M., Muzumdar R.H., Bartke A. (2012). The critical role of metabolic pathways in aging. Diabetes.

[52] Lombardi G., Tauchmanova L., Di Somma C., Musella T., Rota F., Savanelli M.C., Colao A. (2005). Somatopause: Dismetabolic and bone effects. J. Endocrinol. Invest..

[53] O’Neill C., Kiely A.P., Coakley M.F., Manning S., Long-Smith C.M. (2012). Insulin and IGF-1 signalling: Longevity, protein homoeostasis and Alzheimer’s disease. Biochem. Soc. Trans..

[54] Barbieri M., Bonafe M., Franceschi C., Paolisso G. (2003). Insulin/IGF-I-signaling pathway: An evolutionarily conserved mechanism of longevity from yeast to humans. Am. J. Physiol. Endocrinol. Metab..

[55] Lopez-Otin C., Blasco M.A., Partridge L., Serrano M., Kroemer G. (2013). The hallmarks of aging. Cell.

[56] Collino S., Montoliu I., Martin F.P., Scherer M., Mari D., Salvioli S., Bucci L., Ostan R., Monti D., Biagi E. (2013). Metabolic signatures of extreme longevity in northern Italian centenarians reveal a complex remodeling of lipids, amino acids, and gut microbiota metabolism. PLoS ONE.

[57] Montoliu I., Scherer M., Beguelin F., DaSilva L., Mari D., Salvioli S., Martin F.P., Capri M., Bucci L., Ostan R. (2014). Serum profiling of healthy aging identifies phospho- and sphingolipid species as markers of human longevity. Aging.

[58] Bunning B.J., Contrepois K., Lee-McMullen B., Dhondalay G.K.R., Zhang W., Tupa D., Raeber O., Desai M., Nadeau K.C., Snyder M.P. (2020). Global metabolic profiling to model biological processes of aging in twins. Aging Cell.

[59] Park J.S., Kim Y.J. (2020). Anti-aging effect of the ketone metabolite beta-hydroxybutyrate in *Drosophila* intestinal stem cells. Int. J. Mol. Sci..

[60] Ackerman H.D., Gerhard G.S. (2016). Bile acids in neurodegenerative disorders. Front. Aging Neurosci..

[61] Heubi J.E., Setchell K.D., Jha P., Buckley D., Zhang W., Rosenthal P., Potter C., Horslen S., Suskind D. (2015). Treatment of bile acid amidation defects with glycocholic acid. Hepatology.

[62] Schaffer S., Kim H.W. (2018). Effects and mechanisms of taurine as a therapeutic agent. Biomol. Ther..

[63] Pierno S., De Luca A., Camerino C., Huxtable R.J., Camerino D.C. (1998). Chronic administration of taurine to aged rats improves the electrical and contractile properties of skeletal muscle fibers. J. Pharmacol. Exp. Ther..

[64] Vanderschuren H., Boycheva S., Li K.T., Szydlowski N., Gruissem W., Fitzpatrick T.B. (2013). Strategies for vitamin B6 biofortification of plants: A dual role as a micronutrient and a stress protectant. Front. Plant. Sci..

[65] Merigliano C., Mascolo E., Burla R., Saggio I., Verni F. (2018). The relationship between vitamin B6, diabetes and cancer. Front. Genet..

[66] Stover P.J., Field M.S. (2015). Vitamin B-6. Adv. Nutr..

[67] Ruddell R.G., Mann D.A., Ramm G.A. (2008). The function of serotonin within the liver. J. Hepatol..

[68] Cellini B., Zelante T., Dindo M., Bellet M.M., Renga G., Romani L., Costantini C. (2020). Pyridoxal 5’-phosphate-dependent enzymes at the crossroads of host-microbe tryptophan metabolism. Int. J. Mol. Sci..

[69] Michalopoulos G.K., Bhushan B. (2021). Liver regeneration: Biological and pathological mechanisms and implications. Nat. Rev. Gastroenterol. Hepatol..

[70] Kim I.H., Kisseleva T., Brenner D.A. (2015). Aging and liver disease. Curr. Opin. Gastroenterol..

[71] Chen F., Jimenez R.J., Sharma K., Luu H.Y., Hsu B.Y., Ravindranathan A., Stohr B.A., Willenbring H. (2020). Broad distribution of hepatocyte proliferation in liver homeostasis and regeneration. Cell Stem Cell.

[72] Tsuchiya A., Takeuchi S., Watanabe T., Yoshida T., Nojiri S., Ogawa M., Terai S. (2019). Mesenchymal stem cell therapies for liver cirrhosis: MSCs as “conducting cells” for improvement of liver fibrosis and regeneration. Inflamm. Regen..

[73] Payton A., Gibbons L., Davidson Y., Ollier W., Rabbitt P., Worthington J., Pickles A., Pendleton N., Horan M. (2005). Influence of serotonin transporter gene polymorphisms on cognitive decline and cognitive abilities in a nondemented elderly population. Mol. Psychiatry.

[74] Meltzer C.C., Price J.C., Mathis C.A., Butters M.A., Ziolko S.K., Moses-Kolko E., Mazumdar S., Mulsant B.H., Houck P.R., Lopresti B.J. (2004). Serotonin 1A receptor binding and treatment response in late-life depression. Neuropsychopharmacology.

[75] Meltzer C.C., Smith G., DeKosky S.T., Pollock B.G., Mathis C.A., Moore R.Y., Kupfer D.J., Reynolds C.F. (1998). Serotonin in aging, late-life depression, and Alzheimer’s disease: The emerging role of functional imaging. Neuropsychopharmacology.

[76] Grattagliano I., Russmann S., Diogo C., Bonfrate L., Oliveira P.J., Wang D.Q., Portincasa P. (2011). Mitochondria in chronic liver disease. Curr. Drug Targets.

[77] Lee M.J., Jung J., Na K.H., Moon J.S., Lee H.J., Kim J.H., Kim G.I., Kwon S.W., Hwang S.G., Kim G.J. (2010). Anti-fibrotic effect of chorionic plate-derived mesenchymal stem cells isolated from human placenta in a rat model of CCl(4)-injured liver: Potential application to the treatment of hepatic diseases. J. Cell. Biochem..

[78] Kim T.H., Choi J.H., Jun Y., Lim S.M., Park S., Paek J.Y., Lee S.H., Hwang J.Y., Kim G.J. (2018). 3D-cultured human placenta-derived mesenchymal stem cell spheroids enhance ovary function by inducing folliculogenesis. Sci. Rep..

[79] Triant D.A., Whitehead A. (2009). Simultaneous extraction of high-quality RNA and DNA from small tissue samples. J. Hered..

[80] Walker J.A., Kilroy G.E., Xing J., Shewale J., Sinha S.K., Batzer M.A. (2003). Human DNA quantitation using Alu element-based polymerase chain reaction. Anal. Biochem..

[81] Lee M.S., Han H.J., Han S.Y., Kim I.Y., Chae S., Lee C.S., Kim S.E., Yoon S.G., Park J.W., Kim J.H. (2018). Loss of the E3 ubiquitin ligase MKRN1 represses diet-induced metabolic syndrome through AMPK activation. Nat. Commun..

