# Supplementary material for: Metabolic Rewiring by Human Placenta-Derived Mesenchymal Stem Cell Therapy Promotes Rejuvenation in Aged Female Rats
Source: Int J Mol Sci. 2022 Jan 5;23(1):566. doi: 10.3390/ijms23010566 (PMC8745533; doi:10.3390/ijms23010566)
Supplement: Supplementary file 1 [file ijms-23-00566-s001.zip › ijms-1479693-supplementary/Supplementary Figure S1 & Table S2.pdf]

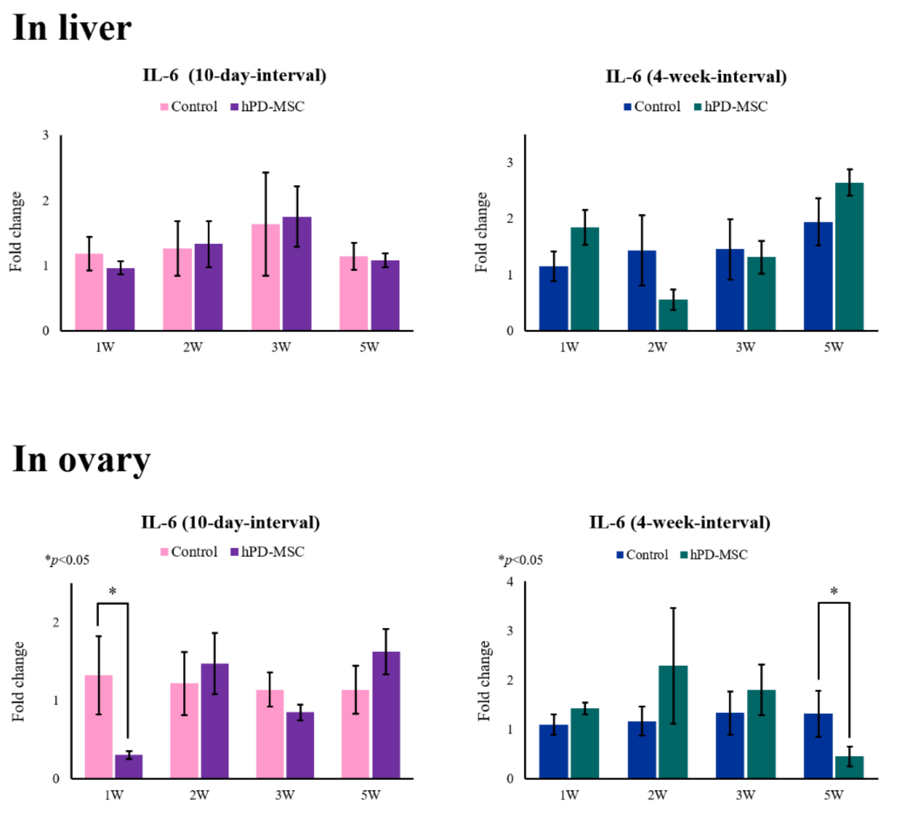

Supplementary Figure S1. hPD-MSC therapy did not trigger the immune response in aged rats. IL-6 expression in the liver was not significantly different between the control and therapy groups. In the ovary, the levels of IL-6 markedly decreased 1 week after hPD-MSC therapy at 10-day intervals and 5 weeks after hPD-MSC therapy at 4-week intervals. The data are presented as the mean ± SEM. The asterisk represents statistical significance at $p < 0.05$. Control, PBS-injected group; hPD-MSCs, multiple-injection hPD-MSC therapy group.

Supplementary Table S2. Primer sequences and RT-PCR conditions.

Gene Symbol	Description	Accession Number	Primer Sequence [a]
Ddc	Dopa decarboxylase	NM_001270853.1	For-TTTTGACTGCTCTGCCATGT<br>Rev-TGAGACAGCTTCACGTGCTT
Hgf	Hepatocyte growth factor	NM_017017.2	For-TGGACCTGAAGGCTCAGATT<br>Rev-TGGTGCTGACTGCATTTCTC
Htr2a	5-Hydroxytryptamine receptor 2a	NM_017254.1	For-GTCCATCAGCAATGAGCAAA<br>Rev-TGGATTGACAGCTGAGGAGA
Tph1	Tryptophan hydroxylase 1	NM_001100634.3	For-AAGATGGGCAGCTGAGAGTC<br>Rev-CGGTTTTGGCAAATTCTCTC
Vegfa	Vascular endothelial growth factor a	NM_031836	For-ATCTTCAAGCCGTCCTGTGT<br>Rev-GCTGCAGGAAGCTCATCTCT
AluYb8			For-CGAGGCGGGTG-<br>GATCATGAGGT<br>Rev- TCTGTCGCCCAGGCCGGACT

[a] For, forward; Rev, reverse.
